# Feasibility and Performance of a Point-of-Care Hepatitis C RNA Assay in a Community Supervision Cohort

**DOI:** 10.1001/jamanetworkopen.2024.38222

**Published:** 2024-10-07

**Authors:** Leah Harvey, Brendan Jacka, Lauri Bazerman, Aurielle Thomas, Marc Moody, Risha Irvin, Curt G. Beckwith

**Affiliations:** 1 Division of Infectious Diseases, The Warren Alpert School of Medicine at Brown University, Providence, Rhode Island; 2 Division of Infectious Diseases, Department of Medicine, The Miriam Hospital, Providence, Rhode Island; 3 The Kirby Institute, University of New South Wales, Sydney, New South Wales, Australia; 4 Rhode Island Department of Corrections, Pawtucket; 5 Division of Infectious Diseases, Johns Hopkins University School of Medicine, Baltimore, Maryland

## Abstract

This cross-sectional study examines the feasibility and clinical performance of a fingerstick point-of-care hepatitis C virus (HCV) RNA assay in a community supervision (probation or parole) setting.

## Introduction

Progress toward hepatitis C virus (HCV) elimination in the US has been stalled by reliance on a multistep diagnostic algorithm to confirm viremia, reducing access to curative treatment.^1,2^ In populations at highest risk of infection—including people who use drugs and those involved in the carceral system—treatment is often inaccessible owing to substantial barriers along the HCV care cascade.^3^ Point-of-care (POC) HCV RNA testing increases treatment uptake, reduces time to treatment initiation,^2^ and is feasible, acceptable, and cost-effective in carceral populations.^4^ However, such assays were only recently approved for clinical use in the US.^5^ We examined the feasibility and clinical performance of a POC HCV RNA assay (Xpert HCV Viral Load; Cepheid) in a nonclinical justice setting as part of a larger study that assessed HCV testing and linkage to care for this population.^3^

## Methods

This cross-sectional study was approved by the Rhode Island Department of Corrections and the institutional review board of The Miriam Hospital; it followed the STROBE guidelines for cross-sectional studies. Study eligibility for the HCV antibody testing study^3^ was age 18 years or older, English-speaking, active community supervision status (probation or parole), and unknown HCV status or no self-reported prior HCV treatment. Participants provided written informed consent. Demographic information was self-reported by participants, and race and ethnicity data were assessed to characterize potential disparities. Details on the assays used and statistical analysis are shown in the eAppendix in Supplement 1.

## Results

Among 482 participants enrolled in the HCV antibody study,^3^ a convenience sample of 203 participants (median [IQR] age, 38.0 [31.0-48.5] years; 159 men [78.3%]) underwent POC HCV RNA testing. The study sample was racially and ethnically diverse, and approximately one-third had not completed high school or reported homelessness (Table 1).

**Table 1.** Demographic Characteristics of Study Participants

Characteristic	Participants, No. (%) (N = 203)
Age, median (IQR), y	38.0 (31.0-48.5)
Gender identity	
Woman	43 (21.2)
Man	159 (78.3)
Transgender	1 (0.5)
Missing	0
Ethnicity	
Hispanic	44 (21.7)
Non-Hispanic	159 (78.3)
Missing	0
Race^a^	
African American or Black	45 (22.1)
Asian	3 (1.5)
Native American or American Indian	17 (8.4)
Two or more races	20 (9.8)
White	97 (47.7)
Missing	5 (2.5)
Other	16 (7.9)
Education	
Less than high school	67 (33.0)
High school	96 (47.3)
More than high school	40 (19.7)
Missing	0
Experiencing homelessness	
No	132 (65.0)
Yes	69 (34.0)
Missing	2 (1.0)
Health insurance^b^	
None	15 (7.4)
Public	177 (87.2)
Private	11 (5.4)
Other	3 (1.5)
Missing or do not know	12 (5.9)

^a^ Data reflect self-reported racial identity. Other category includes Native Hawaiian, Pacific Islander, and participants who reported Hispanic ethnicity and did not identify with any of the listed race categories.

^b^ Categories are not mutually exclusive.

In total, 190 participants (94%) had valid laboratory HCV RNA results; missing data were due to inconclusive results (1 participant), unavailable results (5 participants), or testing was not performed (7 participants). Using laboratory HCV RNA testing for confirmation, the sensitivity of the POC assay for HCV RNA detection was 96.8% (95% CI, 83.3%-99.9%), and the specificity was 99.4% (95% CI, 96.6%-100%) (Table 2). Two participants had discrepant RNA results: one had reactive HCV antibody, POC HCV RNA detected, and laboratory HCV RNA not detected (interpreted as a false-positive POC HCV RNA); and the other had nonreactive HCV antibody, POC HCV RNA not detected, and laboratory HCV RNA detected (interpreted as a false-negative POC HCV RNA, acute HCV infection). Approximately 9 of 39 participants (23.1%) with reactive HCV antibody did not have detectable HCV RNA (POC and laboratory), suggesting spontaneous HCV clearance. One participant had negative HCV antibody and detectable HCV RNA (POC and laboratory) consistent with acute HCV infection.

**Table 2.** Sensitivity and Specificity of Point-of-Care Fingerstick Assay for HCV RNA Detection vs Laboratory-Based Confirmatory Assay

Point-of-care fingerstick HCV RNA assay	Laboratory-based confirmatory HCV RNA test		
	Detected	Not detected	Total
Detected	30	1	31
Not detected	1	158	159
Total	31	159	190

Abbreviation: HCV, hepatitis C virus.

## Discussion

This cross-sectional study demonstrated the feasibility of a POC HCV RNA assay in a nonclinical setting for a population at high risk of HCV infection and confirmed the clinical performance of the fingerstick assay in a nontraditional setting.^6^ Availability of a POC HCV RNA assay for clinical use would have enabled 30 participants, including 2 with acute HCV infection, to have immediate HCV diagnosis. However, 1 participant with acute HCV infection was not identified by the POC HCV RNA assay. Study limitations include a single site that may not be generalizable to other settings, the POC assay was not approved for clinical use when the study was conducted, and laboratory-based confirmatory testing was completed through referral and, therefore, could have been delayed.

Multistep diagnostic algorithms cause unnecessary delay in HCV diagnosis, particularly for marginalized populations.^3,4^ The recent approval of the first POC HCV RNA assay in the US will facilitate same-day initiation of curative HCV treatment in low-barrier, nontraditional settings.^5^ However, the goals of HCV elimination in the US will remain elusive until such assays are widely accessible.

## Notes

Supplement 1. **eAppendix.** Supplemental Methods

Supplement 2.
Data Sharing Statement
