# Supplementary material for: Feasibility and Performance of a Point-of-Care Hepatitis C RNA Assay in a Community Supervision Cohort
Source: JAMA Netw Open. 2024 Oct 7;7(10):e2438222. doi: 10.1001/jamanetworkopen.2024.38222 (PMC11459245; doi:10.1001/jamanetworkopen.2024.38222)
Supplement: Supplement 1. — eAppendix. Supplemental Methods [file jamanetwopen-e2438222-s001.pdf]

Supplemental Online Content

Harvey L, Jacka B, Bazerman L, et al. Feasibility and performance of a point-of-care hepatitis C RNA assay in a community supervision cohort. *JAMA Netw Open*. 2024;7(10):e2438222. doi:10.1001/jamanetworkopen.2024.38222

eAppendix. Supplemental Methods

This supplemental material has been provided by the authors to give readers additional information about their work.

© 2024 Harvey L et al. *JAMA Network Open.*

eAppendix. Supplemental Methods

Patients were offered voluntary POC HCV RNA testing (research-use only; Xpert HCV Viral Load; Cepheid; lower limit of detection [LoD], 22 IU/mL) using a capillary fingerstick blood specimen and were immediately referred for confirmatory laboratory-based qualitative HCV RNA testing (COBAS HCV for 5800/6800/8800 Systems; LoD, 12 IU/mL; or AmpliPrep/COBAS TaqMan HCV v2.0; LoD, 15 IU/mL; both from Roche Diagnostics). Sensitivity and specificity were assessed using the Clopper-Pearson exact method (StataNow/SE statistical software version 18.5; StataCorp). Data were collected from April 2018 to March 2020; analyses were performed in December 2023.

© 2024 Harvey L et al. *JAMA Network Open.*
