# Supplementary material for: Feasibility and Performance of a Point-of-Care Hepatitis C RNA Assay in a Community Supervision Cohort
Source: JAMA Netw Open. 2024 Oct 7;7(10):e2438222. doi: 10.1001/jamanetworkopen.2024.38222 (PMC11459245; doi:10.1001/jamanetworkopen.2024.38222)
Supplement: Supplement 2. — Data Sharing Statement [file jamanetwopen-e2438222-s002.pdf]

Data Sharing Statement

Harvey. Feasibility and Performance of a Point-of-Care Hepatitis C RNA Assay in a Community Supervision Cohort. *JAMA Netw Open*. Published October 07, 2024.
doi:10.1001/jamanetworkopen.2024.38222

Data
Data available: Yes
Data types: Deidentified participant data, Data dictionary
How to access data: LHarvey@Lifespan.org
When available: With publication

Supporting Documents
Document types: None

Additional Information
Who can access the data: Policymakers and researchers whose proposed use of the data has been approved
Types of analyses: For approved purposes
Mechanisms of data availability: With investigator support
